# Red cell distribution width and homocysteine act as independent risk factors for cardiovascular events in newly diagnostic essential hypertension

**DOI:** 10.18632/oncotarget.21964

**Published:** 2017-10-21

**Authors:** Lian-Man He, Chuan-Yu Gao, Yong Wang, Hao Wang, Hai-Ying Zhao

**Affiliations:** ^1^ Department of Hypertension, Henan province People's Hospital, Zheng Zhou, 450003, China; ^2^ Department of Cardiology, Henan province People's Hospital, Zheng Zhou, 450003, China

**Keywords:** red cell distribution width, homocysteine, essential hypertension, cardiovascular event, risk factors

## Abstract

Hyperhomocysteinemia and increased red cell distribution width (RDW) are associated with a higher possibility of adverse clinical outcomes of hypertension. The study aims to validate the effect of homocysteine (Hcy) and RDW on cardiovascular events (CVE) and investigate whether RDW is independently associated with serum Hcy in patients with essential hypertension (EH). The study reviewed 804 patients with newly diagnosed EH in our hospital. The clinical characteristics and laboratory results of all subjects were grouped according to the presence/absence of CVE. Patients in the CVE group had higher RDW and Hcy, as compared to the patients in the no CVE group. Multiple Cox regression analysis demonstrated that both RDW (HR = 1.24, 95% CI =1.02–1.56, *P* = 0.002) and Hcy (HR = 1.37, 95% CI = 1.02–1.80, *P* < 0.001) resulted significantly related to the CVE. Subsequent analysis found that patients with high RDW had higher Hcy levels as compared with those with low RDW (*P* = 0.007). Although Pearson's correlation suggested that RDW was positively correlated with Hcy (r = 0.122, *P* = 0.028), no significant correlation was observed between RDW and Hcy (β = 0.15, *p* = 0.126) after adjusted for a series of potential confounders using multiple linear regression analysis. In conclusion, RDW is not correlated with Hcy in patients with EH. Both RDW and Hcy are independent risk factors for CVE in newly diagnostic EH and have the potential to improve risk stratification.

## INTRODUCTION

Hypertension has been identified as an independent risk factor of cardiovascular diseases, heart failure, arrhythmias, stroke and renal disease, and is the number one cause of deaths all over the world [1]. The etiology of the essential hypertension (EH) includes both genetic and environmental factors [2]. Actually, only a small number of hypertensive patients have an elevated blood pressure alone, with the majority exhibiting additional cardiovascular risk factors, such as obesity [3], dyslipidemia [4], and hyperuricemia [5]. It was worth noting that about 75% of hypertensive patients exhibit high homocysteine (Hcy) in China [6] and several studies have revealed that hyperhomocysteinemia interacts with hypertension in significantly increasing the risk of cardiovascular events (CVE) [7]. Elevated Hcy diminishes the vasodilation of nitric oxide, increases oxidative stress, stimulates the proliferation of vascular smooth muscle cells, lead to vascular constriction, and alters the elastic properties of the vascular wall [8–10]. In the last decades, researches have indicated a link between elevated Hcy and increased mortality among hypertensive patients [11–13]. However, limited studies have examined the association between Hcy and CVE in newly diagnosed hypertensive patients without cardiovascular comorbidities [14].

Red cell distribution width (RDW) is a component of the complete blood cell count [15]. Used in clinical setting as a tool for the differential diagnosis of anemia, recent studies have shown RDW is a predictor of mortality in multiple clinical conditions [16–18]. High RDW was independently associated with presence of coronary artery disease and a graded independent correlation between RDW values and risk of CVE were observed [19, 20]. RDW may also be a helpful diagnostic and prognostic indicator for acute coronary syndromes, heart failure, metabolic syndrome and obstructive sleep apnea [21]. Importantly, higher RDW values have been found in patients with prehypertension and hypertension compared with normotensives [16]. However, to date, whether RDW has a prognostic value in EH is unclear. A recent study also identified a close relationship between RDW and Hcy in health check-up adults [22]. Considering the above situations, we aimed to observe the effect of Hcy and RDW on CVE, meanwhile to investigate any existing relationship between RDW and Hcy, two conventional and inexpensive indexes, in newly diagnosed and untreated patients with EH.

## RESULTS

### Study population and survival analysis

This study comprised 419 men and 385 women with a mean age of 46.3 ± 10.9 years. Baseline clinical and laboratory characteristics of all patients were divided according to the presence of CVE and were shown in Table 1. During the follow-up (4.2 ± 1.1 years), 185 patients experienced CVE in our cohort. Among these, 24 patients (12.9%) died because of CVE as previously described, and 115 patients had coronary events (62.2%), the remaining 46 patients (24.9%) experienced cerebrovascular events. Older patients were more liable to experience CVE (*P* = 0.006). Patients in the CVE group had higher systolic blood pressure (SBP) (*P* < 0.001), higher creatinine (*P* = 0.028), uric acid (*P* = 0.016), Hcy (*P* < 0.001), hemoglobin levels (*P* = 0.013) and RDW values (*P* < 0.001), as compared to the patients in no CVE group (Table 1). However, no significant differences in patients’ sex, body mass index, heart rate, smoking, alcohol, and other laboratory values such as triglyceride, total cholesterol and BUN were observed between above two groups. On multiple Cox regression analysis, levels of Hcy (3 μmol/L increase in Hcy, HR = 1.36, 95% CI = 1.01–1.80, *P* < 0.001, Table 2-Model 3) and RDW (1 % increase in RDW, HR = 1.24, 95% CI = 1.02–1.56, *P* = 0.002, Table 2-Model 3) were all significantly correlated to the incidence rate of CVE. We also found SBP, LDL-C and uric acid were significant biomarkers for CVE in newly diagnostic EH patients.

**Table 1:** Baseline demographic, clinical characteristics and laboratory results of the study groups, divided into two groups according to the presence of cardiovascular events

Clinical Characteristics	No CVE (*n* = 619)	CVE (*n* = 185)	*P* value
Age (years)	45.1 ± 9.11	47.1 ± 7.32	0.006
Male (*n*, %)	321(51.9)	98 (53.0)	0.246
BMI (kg/m^2^)	26.0 ± 3.17	26.4 ± 3.82	0.152
Heart rate (bpm)	78 ± 14	80 ± 15	0.094
Smoking (*n*, %)	102 (16.5)	40 (21.6)	0.134
Alcohol (%)	204 (32.9)	75 (40.5)	0.070
SBP (mmHg)	150 ± 12	157 ± 13	< 0.001
DBP (mmHg)	87 ± 9	90 ± 9	0.001
Triglyceride (mmol/L)	1.47 ± 0.25	1.50 ± 0.31	0.177
Total cholesterol (mmol/L)	5.17 ± 0.62	5.24 ± 0.69	0.190
HDL-C (mmol/L)	1.29 ± 0.26	1.27 ± 0.25	0.355
LDL-C (mmol/L)	2.92 ± 0.34	2.95 ± 0.30	0.280
FBG (mmol/L)	5.64 ± 0.45	5.67 ± 0.49	0.436
Creatinine (μmol/L)	76.5 ± 12.5	78.9 ± 14.8	0.028
BUN (mmol/L)	4.59 ± 0.70	4.62 ± 0.84	0.626
Uric acid (μmol/L)	285 ± 53.7	296 ± 57.8	0.016
Hcy (μmol/L)	7.86 ± 2.55	12.53 ± 4.01	< 0.001
WBC count (103/mm^3^)	6.48 ± 0.89	6.51 ± 0.90	0.688
RBC count (103/mm^3^)	4.96 ± 0.51	5.02 ± 0.56	0.170
Hemoglobin (g/L)	149 ± 14	152 ± 16	0.013
Plt count (103/mm^3^)	238 ± 48	241 ± 53	0.467
RDW (%)	11.2 ± 0.88	12.5 ± 0.94	< 0.001
Hs-CRP (mg/dL)	0.39 ± 0.12	0.40 ± 0.14	0.339

Note: CVE = cardiovascular events; BMI = body mass index; SBP = systolic blood pressure; DBP = diastolic blood pressure; HDL-C = high-density lipoprotein cholesterol; LDL-C = low-density lipoprotein cholesterol; FBG = fasting blood glucose; BUN = blood urea nitrogen; WBC = white blood cell; RBC = red blood cell; Plt = blood platelet; Hs-CRP = high-sensitivity C-reactive protein.

**Table 2:** Cox regression model of increasing complexity on cardiovascular events (*n* = 804)

	HR (95% CI)		
Variable units of increase	Model 1	Model 2	Model 3
Age, one year	1.05 (1.02–1.16)	1.02 (0.96–1.06)	1.03 (0.92–1.04)
	*P* = 0.001	*P* = 0.173	*P* = 0.245
Smoking	1.04 (1.00–1.58)	0.96 (0.94–1.36)	0.95 (0.94–1.34)
	*P* = 0.022	*P* = 0.093	*P* = 0.094
Alcohol	0.95 (0.65–1.05)	0.95 (0.62–1.03)	0.93 (0.60–1.02)
	*P* = 0.203	*P* = 0.205	*P* = 0.210
SBP, 10 mmHg	1.15 (1.02–1.74)	1.11 (1.00–1.56)	1.10 (1.00–1.55)
	*P* = 0.017	*P* = 0.020	*P* = 0.022
LDL-C, 0.2 mmol/L	1.07 (1.01–1.22)	1.04 (1.00–1.17)	1.04 (1.00–1.18)
	*P* = 0.037	*P* = 0.031	*P* = 0.030
Creatinine, 10 μmol/L	0.92 (0.73–1.19)	0.88 (0.69–1.11)	0.88 (0.65–1.09)
	*P* = 0.265	*P* = 0.304	*P* = 0.306
Uric acid, 10 μmol/L	1.21 (1.06–1.77)	1.10 (1.01–1.42)	1.10 (1.01–1.41)
	*P* = 0.009	*P* = 0.012	*P* = 0.011
Hcy, 3 μmol/L		1.37 (1.02–1.80)	1.36 (1.01–1.80)
		*P* < 0.001	*P* < 0.001
RDW, 1%			1.24 (1.02–1.56)
			*P* = 0.002

Note: HR = hazard ratio; SBP = systolic blood pressure; LDL-C = low-density lipoprotein cholesterol.

### Clinical significance of RDW in newly diagnosed hypersensitive patients

To further reveal the clinical significance of RDW in hypersensitive patients, patients were assigned into low RDW group and high RDW group. As illustrated in Table 3, patients with higher RDW values tend to have higher age (*P* = 0.041), BMI (*P* = 0.003) and SBP (*P* = 0.017). With respect to laboratory results, the level of HDL-C in the high RDW group was lower, while the level of LDL-C, uric acid, Hcy, RBC count, and hemoglobin were significantly higher than that in the low RDW group (*P* < 0.05 for all). There were no statistical differences between two groups when comparing to the sex, heart rate, smoking, alcohol, triglyceride, FBG, creatinine, BUN, WBC count, Plt count and hs-CRP.

**Table 3:** Clinical characteristics and laboratory results of the study groups divided into two groups according to the value of RDW

Clinical Characteristics	Low RDW (*n* = 312)	High RDW (*n* = 492)	*P* value
Age (years)	45.3 ± 10.6	46.8 ± 9.81	0.041
Male (*n*, %)	174 (55.8)	245 (49.8)	0.114
BMI (kg/m^2^)	25.4 ± 3.68	26.3 ± 4.40	0.003
Heart rate (bpm)	78 ± 12	77 ± 13	0.274
Smoking (*n*, %)	62(19.9)	80 (16.3)	0.225
Alcohol (%)	97 (31.1)	182 (37.0)	0.102
SBP (mmHg)	148 ± 11	150 ± 12	0.017
DBP (mmHg)	87 ± 10	88 ± 10	0.167
Triglyceride (mmol/L)	1.50 ± 0.29	1.52 ± 0.32	0.371
Total cholesterol (mmol/L)	5.11 ± 0.77	5.17 ± 0.65	0.236
HDL-C (mmol/L)	1.31 ± 0.22	1.27 ± 0.23	0.015
LDL-C (mmol/L)	2.90 ± 0.30	2.96 ± 0.32	0.008
FBG (mmol/L)	5.63 ± 0.44	5.66 ± 0.50	0.386
Creatinine (μmol/L)	77.1 ± 13.3	78.0 ± 14.5	0.418
BUN (mmol/L)	4.62 ± 0.65	4.67 ± 0.72	0.320
Uric acid (μmol/L)	277 ± 50.3	289 ± 69.1	0.003
Hcy (μmol/L)	8.74 ± 3.55	9.49 ± 4.01	0.007
WBC count (103/mm^3^)	6.53 ± 1.03	6.50 ± 0.84	0.652
RBC count (103/mm^3^)	4.94 ± 0.49	5.03 ± 0.61	0.028
Hemoglobin (g/L)	146 ± 16	151 ± 19	< 0.001
Plt count (103/mm^3^)	237 ± 52	240 ± 47	0.398
Hs-CRP (mg/dL)	0.41 ± 0.10	0.42 ± 0.11	0.194

Note: BMI = body mass index; SBP = systolic blood pressure; DBP = diastolic blood pressure; HDL-C = high-density lipoprotein cholesterol; LDL-C = low-density lipoprotein cholesterol; FBG = fasting blood glucose; BUN = blood urea nitrogen; WBC = white blood cell; RBC = red blood cell; Plt = blood platelet; Hs-CRP = high-sensitivity C-reactive protein.

### Correlation between RDW and variables in hypertension

**Figure 1:**
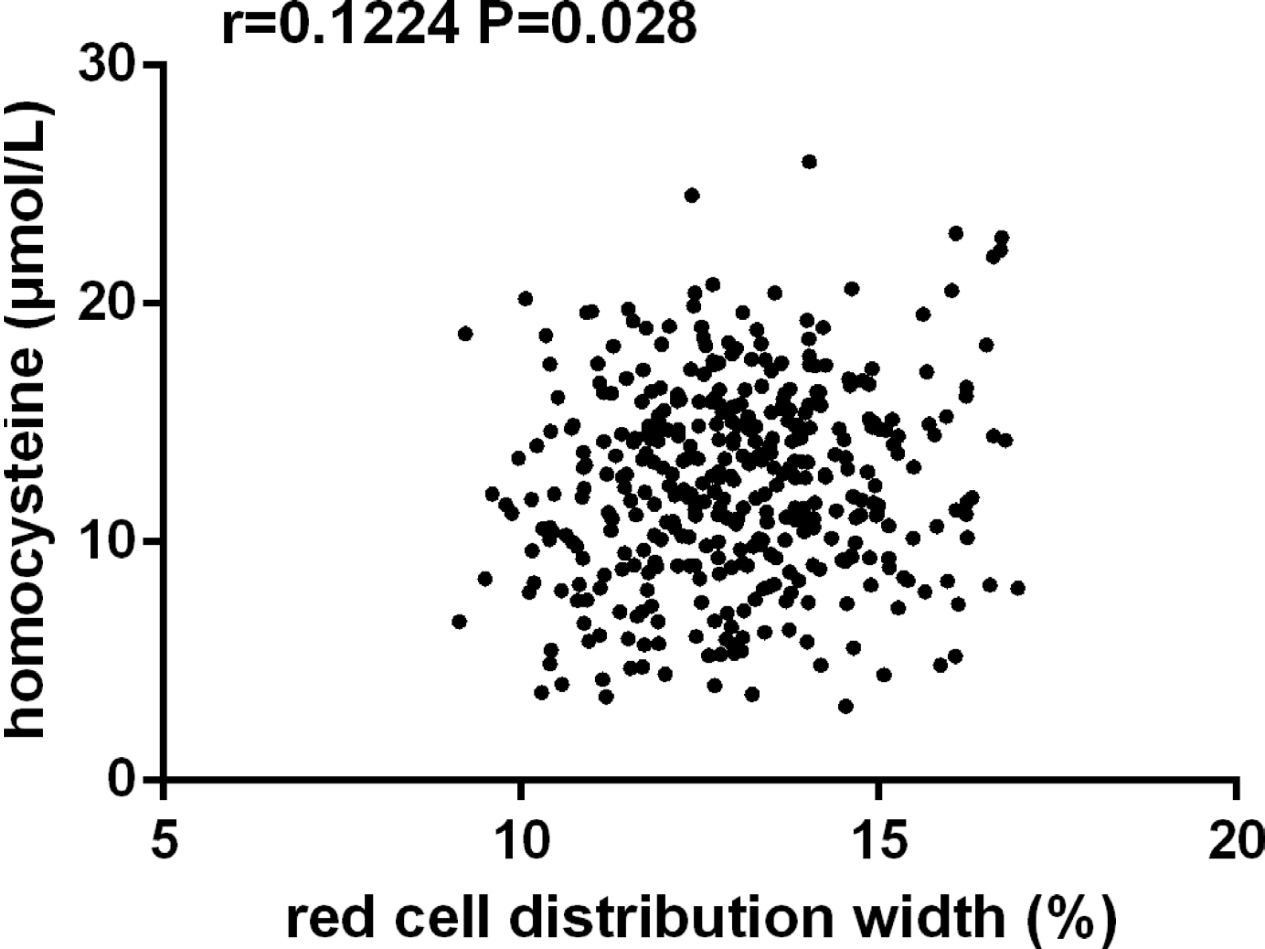
Correlation analysis showed that red cell distribution width was positively correlated with homocysteine levels in patients with essential hypertension (*n* = 804, r = 0.1244, *P* = 0.028)

Correlation analysis identified that RDW values in patients with EH was positively correlated with age (r = 0.117, *P* = 0.036), BMI (r = 0.159, *P* = 0.022), LDL-C (r = 0.155, *P* = 0.026), uric acid (r = 0.178, *P* = 0.010), RBC count (r = 0.202, *P* = 0.004), hemoglobin (r = 0.209, *P* = 0.001), Hcy (r = 0.122, *P* = 0.028, Figure 1) and was negatively associated with HDL-C (r =–0.173, *P* = 0.017). No linear correlation was observed between RDW and variables including age, sex, alcohol, blood pressure, total cholesterol, FBG, WBC count, and hs-CRP (Table 4).

**Table 4:** Correlation analysis between RDW and variables in patients with essential hypertension

Parameters	Correlation coefficient	*P* values
Age (years)	0.117	0.036
Male (*n*, %)	0.074	0.305
BMI (kg/m^2^)	0.159	0.022
Alcohol (%)	0.071	0.316
SBP (mmHg)	0.013	0.665
DBP (mmHg)	–0.004	0.781
Total cholesterol (mmol/L)	0.012	0.677
HDL-C (mmol/L)	–0.173	0.017
LDL-C (mmol/L)	0.155	0.026
FBG (mmol/L)	0.029	0.584
Uric acid (μmol/L)	0.178	0.010
WBC count (103/mm^3^)	0.094	0.191
RBC count (103/mm^3^)	0.202	0.004
Hemoglobin (g/L)	0.209	0.001
Hs-CRP (mg/dL)	0.101	0.070
Hcy	0.122	0.028

Note: BMI = body mass index; SBP = systolic blood pressure; DBP = diastolic blood pressure; HDL-C = high-density lipoprotein cholesterol; LDL-C = low-density lipoprotein cholesterol; FBG = fasting blood glucose; WBC = white blood cell; RBC = red blood cell; Plt = blood platelet; Hs-CRP = high-sensitivity C-reactive protein.

### Multiple analysis for the effect of independent variables on RDW

In multivariate linear regression analysis (Table 5), after adjusting age, sex, alcohol, blood pressure, total cholesterol, HDL-C, FBG, RBC count, and LDL-C, however, the correlation between RDW and Hcy disappeared. RDW was significantly associated with age (β = 0.12, *P* = 0.010), BMI (β = 0.14, *P* = 0.029), and uric acid (β = 0.26, *P* = 0.006).

**Table 5:** Multiple linear regression analysis for the effect of independent variables on RDW

Model		B	SE	t	*P*
1	Constant	7.90	1.20	4.372	< 0.001
	Age	0.12	0.03	2.374	0.018
	BMI	0.16	0.07	1.827	0.037
	LDL-C	0.03	0.01	1.319	0.129
	Hcy	0.16	0.07	1.789	0.041
	Uric acid	0.28	0.05	3.107	0.009
2	Constant	7.63	0.98	4.736	< 0.001
	Age	0.12	0.03	2.374	0.018
	BMI	0.14	0.07	1.827	0.034
	Hcy	0.15	0.07	1.611	0.126
	Uric acid	0.26	0.05	3.146	0.006
3	Constant	7.63	0.97	4.736	< 0.001
	Age	0.12	0.03	2.380	0.010
	BMI	0.14	0.07	1.840	0.029
	Uric acid	0.26	0.04	3.146	0.006

Note: BMI = body mass index; LDL-C = low-density lipoprotein cholesterol.

## DISCUSSION

We found both RDW and Hcy are independent risk factors for CVE in newly diagnosed hypertensive patients in a study with relative large sample size. In addition, we found that patients with higher RDW tend to have higher Hcy concentration. However, RDW was not correlated with Hcy in patients with EH after adjusting for potential confounders including age, sex, BMI, alcohol, blood pressure, total cholesterol, HDL-C, FBG, RBC count, and LDL-C.

A meta-analysis of published epidemiological studies on the relationship between Hcy and hypertension risk showed that elevated Hcy levels increased the risk for EH [23]. In contrast, some studies reported no relationship between Hcy levels and EH risk after adjusting for confounding factors [24, 25] and Hcy was not a causal factor of blood pressure [26]. They suggested Hcy is more likely a marker than a cause of this disease. Findings from clinical trials found that folic acid and B vitamins supplementation decreased Hcy levels but the blood pressure did not change [27, 28]. As to prognostic value, elevated Hcy level has been reported strongly correlated with worse outcome in patients with CAD [29], and it is also an independent predictor of ischemic heart disease in healthy groups [30]. What's more, Veeranna et al demonstrated that elevated Hcy levels have additive prognostic values in terms of predicting CVE in the general population [31]. However, few literatures have determined the association between Hcy and CVE in untreated hypertension. We noticed that a pilot study with 83 subjects demonstrated that Hcy levels may be associated with EH, but was unrelated to prognosis [32]. Another study with larger samples indicated an independent association of Hcy with cerebro-cardiovascular disease in western country [33]. Consistent with previous results, we identified that Hcy was an independent risk factor for CVE in patients with untreated EH, even after adjusting for related confounders.

It is well established that chronic inflammation is at the root of atherosclerosis and its complications. And increasing evidence shows an important relationship among inflammation, oxidative stress and hypertension [34, 35]. RDW is an indicator of red cell size variation called anisocytosis which is calculated by dividing the standard deviation of RBC volume by mean corpuscular volume (MCV) and multiplying by 100 to express the result as a percentage [36]. It is mainly used for the differential diagnosis of microcytic anemia. Recently, similar with Hcy, RDW was found to be associated with morbidity and mortality in cardiovascular diseases [37]. A study showed that RDW may be a predictor marker of persistent pulmonary hypertension of the newborn [38]. In addition, Triantafyllidi et al. demonstrated higher RDW values in non-dippers hypertension compared to dippers and normotensives [39]. We firstly found that high RDW may act as an indicator for CVE in patients with EH. The mechanistic links between RDW and hypertension are not yet fully understood. Current studies hypothesized that this indicative property of RDW maybe result from its reflection an underlying chronic inflammation [19, 40]. A study demonstrated a strong, graded association of RDW with hs-CRP and endoplasmic reticulum stress (ERS) independent of numerous confounding factors in outpatients, which suggesting that increased RDW may be reflective of an inflammatory state that leading to impaired RBC maturation [41]. RDW was shown associated with inflammatory cytokine hs-CRP in patients with coronary artery disease [42]. Nevertheless, hs-CRP showed no correlation with RDW in our study. These may because we analyze their relationship among newly diagnosed untreated hypertensive patients, it is still unclear whether patients with higher RDW had higher inflammatory levels with the progress of hypertension. Besides, a significant correlation between RDW and uric acid was identified (β = 0.178). Uric acid is also act as an independent risk factor of hypertension, according to the previous studies [43, 44], which further validating that elevated RDW should be considered to be a marker of CVE.

Of note, in a recent published paper, researchers suggested that RDW may predict Hcy levels among the health check-up adults without vitamin B12 and folate deficiencies. They identified that RDW was positively correlated with Hcy (r = 0.227) [22]. Inconsistently, we analyzed their relationship among untreated hypertensive patients and found that although the relationship was observed in univariate analysis, no significantly correlation between them after adjusting for confounders. Indeed, the abovementioned study did not point out how many subjects had hypertension, which may the most reason for the discrepancy. Besides, these also suggested other multiple factors between RDW and Hcy in the progress of EH.

Certain limitations of our study should be mentioned. Firstly, we only reviewed the parts of available subjects in our study. This situation may influence the predictor value of RDW and Hcy on CVE and a true prospective study is quite needed. On the other hand, the patients analyzed in this study received no antihypertensive medications before. However, a large clinical study found Hcy levels were 9% higher in those who were using antihypertensive agents than in those who were not and this effect depended on the type of the agents [45]. Whether the correlation between RDW and Hcy exert among treated hypertensive patients is still undefined. Moreover, as the etiology of hypertension includes numerous genetic, environmental, racial, and regional factor, our single-central analysis could not take all above factors into account. Further studies with subgroup analysis are required to confirm the effects of RDW and Hcy on CVE and explored exact relationship between them in the clinical settings.

## MATERIALS AND METHODS

### Study design and population

From March 2008 to June 2012, 4000 consecutive patients admitted to the department of hypertension in Henan province People's Hospital were screened for analysis. Diagnosis of hypertension was established in all patients according to current guidelines [46]. High blood was defined as systolic blood pressure (SBP) of ≥ 140 mmHg and/or diastolic blood pressure (DBP) of ≥ 90 mmHg. Blood pressure was obtained by an automated device (Omron HEM-8732T, Omron Healthcare Co. Ltd, Kyoto, Japan) after each subject had been supine for 15 min and the average of three readings was recorded. Exclusion criteria was (1) ever or current use of antihypertensive medications, patients with hypertensive emergencies or secondary hypertension; (2) coronary artery disease, congenital heart disease, cardiomyopathy, congestive heart failure, and primary valvular disease; (3) second-and third-degree AV block, atrial flutter/fibrillation, and pacemaker rhythm; (4) patients younger than 18 years and older than 80 years and pregnant women were also excluded; (5) symptomatic peripheral vascular disease, diabetes mellitus, thyroid disease, autoimmune disease, chronic kidney diseases, hematologic disease, malignant tumor or other severe disease. Finally, 2361 patients meet above criterion and all patients received regular antihypertensive treatment in accordance with the Chinese guidelines for the management of hypertension [46]. After then, patients were followed up for any incidence of CVE, including cardiac death, angina, myocardial infarction, coronary revascularization, admission due to heart failure and stroke, peripheral arterial disease, and transient ischemic attack [47], until December 2016. The study protocol was approved by the Research Ethics Committee of the Henan province People's Hospital.

**Figure 2:**
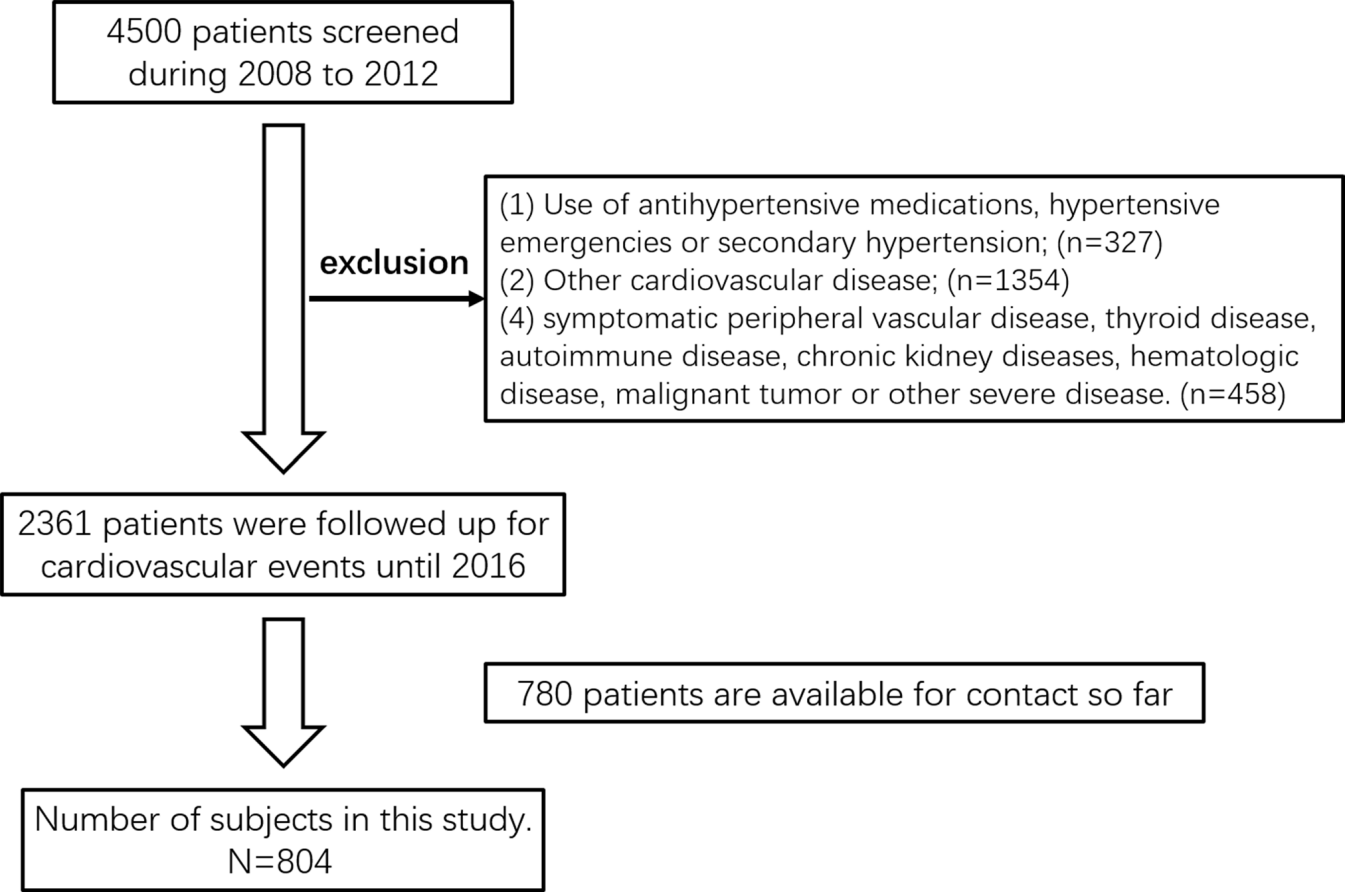
Flow diagram of the phases of the study

So far, 780 patients were available for contact, and among the following up patients, 24 patients were dead due to CVE (other causes of mortality were not considered). These two parts subjects were selected for retrospectively analysis in the current study (Figure 2). Characteristics containing age, sex, body mass index (BMI), heart rate, smoking, alcohol status and laboratory tests were reviewed. Smokers were defined as at least 5 years smoking history and up to 1 year before the study. Drinkers were defined as drinking at least two times a week and lasting at least for 1 year. Patients were assigned to two groups, CVE and non-CVE group for analyzing risk factors.

### Laboratory measurements

Blood samples were drawn before any medical intervention from all subjects into ethylene diamine tetraacetic acid (EDTA) tubes on the morning after a 12-h fast to measure concentrations of the following parameters: Hcy, triglyceride, total cholesterol, high-density lipoprotein cholesterol (HDL-C), low-density lipoprotein cholesterol (LDL-C), fasting blood glucose (FBG), creatinine, blood urea nitrogen (BUN), uric acid, and blood routine test, which includes white blood cell (WBC), red blood cell (RBC), hemoglobin and RDW. Blood routine test was determined using automated hematology analyzers. Other biochemical parameters were measured in the laboratory of Hospital using automatic biochemistry analyzer/bench-top ACCENT-200 (Carolina Liquid Chemistries Corp, Greensboro, NC, USA).

### Statistical analysis

The data were analyzed using SPSS version 22.0 (SPSS Inc., Chicago, IL, USA). Continuous variables were presented as medians ± standard deviation and categorical variables were expressed as frequencies with percentages. The effect of RDW for explaining the incidence rate of study outcomes was investigated by multiple Cox regression analyses. We tested a series of factors including age, smoking, alcohol, SBP, LDL-C, creatinine, uric acid, Hcy and RDW in multiple Cox models. All patients were divided into two groups presence/absence of CVE, or low RDW group and high RDW group according to median (12.5%). The groups were compared using the Student's *t*-test or chi-square analysis. Pearson's correlation or Spearman rank correlation coefficients were calculated between RDW and other variables. Univariable and multivariable linear regression were used to identify significant determinants for RDW, with adjustments for variables related to increased Hcy, including age, sex, BMI, heart rate, smoking and BUN. A *P*-value of < 0.05 was considered statistically significant.

## References

[1] Blacher J, Levy BI, Mourad JJ, Safar ME, Bakris G (2016). From epidemiological transition to modern cardiovascular epidemiology: hypertension in the 21st century. Lancet.

[2] Xi B, Cheng H, Shen Y, Zhao X, Hou D, Wang X, Mi J (2012). Physical activity modifies the associations between genetic variants and hypertension in the Chinese children. Atherosclerosis.

[3] Patel SA, Deepa M, Shivashankar R, Ali MK, Kapoor D, Gupta R, Lall D, Tandon N, Mohan V, Kadir MM, Fatmi Z, Prabhakaran D, Narayan KM (2017). Comparison of multiple obesity indices for cardiovascular disease risk classification in South Asian adults: the CARRS Study. PLoS One.

[4] Kotsis V, Antza C, Doundoulakis I, Stabouli S (2017). Markers of early vascular ageing. Curr Pharm Des.

[5] Perticone M, Tripepi G, Maio R, Cimellaro A, Addesi D, Baggetta R, Sciacqua A, Sesti G, Perticone F (2017). Risk reclassification ability of uric acid for cardiovascular outcomes in essential hypertension. Int J Cardiol.

[6] Chang Y, Li Y, Guo X, Chen Y, Dai D, Sun Y (2017). The Prevalence of Hypertension Accompanied by High Homocysteine and its Risk Factors in a Rural Population: A Cross-Sectional Study from Northeast China. Int J Environ Res Public Health.

[7] Momin M, Jia J, Fan F, Li J, Dou J, Chen D, Huo Y, Zhang Y (2017). Relationship between plasma homocysteine level and lipid profiles in a community-based Chinese population. Lipids Health Dis.

[8] Meng L, Liu L, Zhou C, Pan S, Zhai X, Jiang C, Guo Y, Ji Z, Chi J, Peng F, Guo H (2016). Polyphenols and Polypeptides in Chinese Rice Wine Inhibit Homocysteine-induced Proliferation and Migration of Vascular Smooth Muscle Cells. J Cardiovasc Pharmacol.

[9] Toda N, Okamura T (2016). Hyperhomocysteinemia impairs regional blood flow: involvements of endothelial and neuronal nitric oxide. Pflugers Arch.

[10] Liu J, Liu H, Zhao H, Zhou Y, Li L, Wang H (2016). Relationship between cardio-ankle vascular index and homocysteine in hypertension subjects with hyperhomocysteinemia. Clin Exp Hypertens.

[11] Rossi GP, Maiolino G, Seccia TM, Burlina A, Zavattiero S, Cesari M, Sticchi D, Pedon L, Zanchetta M, Pessina AC (2006). Hyperhomocysteinemia predicts total and cardiovascular mortality in high-risk women. J Hypertens.

[12] McCully KS (2015). Homocysteine and the pathogenesis of atherosclerosis. Expert Rev Clin Pharmacol.

[13] Li J, Jiang S, Zhang Y, Tang G, Wang Y, Mao G, Li Z, Xu X, Wang B, Huo Y (2015). H-type hypertension and risk of stroke in chinese adults: A prospective, nested case-control study. J Transl Int Med.

[14] Xu B, Kong X, Xu R, Song Y, Liu L, Zhou Z, Gu R, Shi X, Zhao M, Huang X, He M, Fu J, Cai Y (2017). Homocysteine and all-cause mortality in hypertensive adults without pre-existing cardiovascular conditions: effect modification by MTHFR C677T polymorphism. Medicine (Baltimore).

[15] Buttarello M, Plebani M (2008). Automated blood cell counts: state of the art. Am J Clin Pathol.

[16] Tanindi A, Topal FE, Topal F, Celik B (2012). Red cell distribution width in patients with prehypertension and hypertension. Blood Press.

[17] Ozcan F, Turak O, Durak A, Işleyen A, Uçar F, Giniş Z, Uçar F, Başar FN, Aydoğdu S (2013). Red cell distribution width and inflammation in patients with non-dipper hypertension. Blood Press.

[18] Yılmaz ZV, Yılmaz E, Küçüközkan T (2016). Red blood cell distribution width: A simple parameter in preeclampsia. Pregnancy Hypertens.

[19] Tonelli M, Sacks F, Arnold M, Moye L, Davis B, Pfeffer M, for the Cholesterol and Recurrent Events (CARE) Trial Investigators (2008). Relation Between Red Blood Cell Distribution Width and Cardiovascular Event Rate in People With Coronary Disease. Circulation.

[20] Karaçağlar E, Bal U, Hasırcı S, Yılmaz M, Doğanözü E, Coşkun M, İ Atar, Yıldırır A, Müderrisoğlu H (2016). Coronary artery disease detected by coronary computed tomography angiography is associated with red cell distribution width. Turk Kardiyol Dern Ars.

[21] Salvagno GL, Sanchis-Gomar F, Picanza A, Lippi G (2015). Red blood cell distribution width: A simple parameter with multiple clinical applications. Crit Rev Clin Lab Sci.

[22] Peng YF, Pan GG (2017). Red blood cell distribution width predicts homocysteine levels in adult population without vitamin B12 and folate deficiencies. Int J Cardiol.

[23] Zhong F, Zhuang L, Wang Y, Ma Y (2017). Homocysteine levels and risk of essential hypertension: A meta-analysis of published epidemiological studies. Clin Exp Hypertens.

[24] Sundström J, Sullivan L, D’Agostino RB, Jacques PF, Selhub J, Rosenberg IH, Wilson PW, Levy D, Vasan RS (2003). Plasma homocysteine, hypertension incidence, and blood pressure tracking: the Framingham Heart Study. Hypertension.

[25] Fakhrzadeh H, Ghotbi S, Pourebrahim R, Heshmat R, Nouri M, Shafaee A, Larijani B (2005). Plasma homocysteine concentration and blood pressure in healthy Iranian adults: the Tehran Homocysteine Survey (2003-2004). J Hum Hypertens.

[26] Borges MC, Hartwig FP, Oliveira IO, Horta BL (2016). Is there a causal role for homocysteine concentration in blood pressure? A Mendelian randomization study. Am J Clin Nutr.

[27] Bønaa KH, Njølstad I, Ueland PM, Schirmer H, Tverdal A, Steigen T, Wang H, Nordrehaug JE, Arnesen E, Rasmussen K, NORVIT Trial Investigators (2006). Homocysteine lowering and cardiovascular events after acute myocardial infarction. N Engl J Med.

[28] Lonn E, Yusuf S, Arnold MJ, Sheridan P, Pogue J, Micks M, McQueen MJ, Probstfield J, Fodor G, Held C, Genest J, Outcomes Heart, Prevention Evaluation (HOPE) 2 Investigators (2006). Homocysteine lowering with folic acid and B vitamins in vascular disease. N Engl J Med.

[29] Kwon SW, Kim JY, Suh YJ, Lee DH, Yoon YW, Lee BK, Jung YH, Choi EY, Hong BK, Rim SJ, Kwon HM (2016). Prognostic Value of Elevated Homocysteine Levels in Korean Patients with Coronary Artery Disease: A Propensity Score Matched Analysis. Korean Circ J.

[30] Homocysteine Studies Collaboration (2002). Homocysteine and risk of ischemic heart disease and stroke: a meta-analysis. JAMA.

[31] Veeranna V, Zalawadiya SK, Niraj A, Pradhan J, Ference B, Burack RC, Jacob S, Afonso L (2011). Homocysteine and reclassification of cardiovascular disease risk. J Am Coll Cardiol.

[32] Lip GY, Edmunds E, Martin SC, Jones AF, Blann AD, Beevers DG (2001). A pilot study of homocyst(e)ine levels in essential hypertension: relationship to von Willebrand factor, an index of endothelial damage. Am J Hypertens.

[33] Catena C, Colussi G, Nait F, Capobianco F, Sechi LA (2015). Elevated Homocysteine Levels Are Associated With the Metabolic Syndrome and Cardiovascular Events in Hypertensive Patients. Am J Hypertens.

[34] Agita A, Alsagaff MT (2017). Inflammation, Immunity, and Hypertension. Acta Med Indones.

[35] Guzik TJ, Touyz RM (2017). Oxidative Stress, Inflammation, and Vascular Aging in Hypertension. Hypertension.

[36] Evans TC, Jehle D (1991). The red blood cell distribution width. J Emerg Med.

[37] Xanthopoulos A, Giamouzis G, Tryposkiadis K, Paraskevopoulou E, Paraskevopoulou P, Karagiannis G, Patsilinakos S, Parissis J, Farmakis D, Butler J, Skoularigis J, Triposkiadis F (2017). A simple score for early risk stratification in acute heart failure. Int J Cardiol.

[38] Sagheb S, Sepidarkish M, Mohseni SO, Movahedian A, Mosayebi Z (2017). Red Cell Distribution Width as a Predictor of Persistent Pulmonary Hypertension of the Newborn. Am J Perinatol.

[39] Triantafyllidi H, Palaiodimos L, Ikonomidis I, Schoinas A, Pavlidis G, Trivilou P, Lekakis J (2016). The independent association of two “priceless” parameters: pulse pressure and red cell distribution width in recently diagnosed hypertensive patients. Hellenic J Cardiol.

[40] Semba RD, Patel KV, Ferrucci L, Sun K, Roy CN, Guralnik JM, Fried LP (2010). Serum antioxidants and inflammation predict red cell distribution width in older women. the Women's Health and Aging Study I. Clin Nutr.

[41] Lippi G, Targher G, Montagnana M, Salvagno GL, Zoppini G, Guidi GC (2009). Relation between red blood cell distribution width and inflammatory biomarkers in a large cohort of unselected outpatients. Arch Pathol Lab Med.

[42] Lappé JM, Horne BD, Shah SH, May HT, Muhlestein JB, Lappé DL, Kfoury AG, Carlquist JF, Budge D, Alharethi R, Bair TL, Kraus WE, Anderson JL (2011). Red cell distribution width, C-reactive protein, the complete blood count, and mortality in patients with coronary disease and a normal comparison population. Clin Chim Acta.

[43] Masuo K, Kawaguchi H, Mikami H, Ogihara T, Tuck ML (2003). Serum uric acid and plasma norepinephrine concentrations predict subsequent weight gain and blood pressure elevation. Hypertension.

[44] Luo M, Li ZZ, Li YY, Chen LZ, Yan SP, Chen P, Hu YY (2014). Relationship between red cell distribution width and serum uric acid in patients with untreated essential hypertension. Sci Rep.

[45] Jacques PF, Bostom AG, Wilson PW, Rich S, Rosenberg IH, Selhub J (2001). Determinants of plasma total homocysteine concentration in the Framingham Offspring cohort. Am J Clin Nutr.

[46] Liu LS, Writing Group of 2010 Chinese Guidelines for the Management of Hypertension (2011). [2010 Chinese guidelines for the management of hypertension. Zhonghua Xin Xue Guan Bing Za Zhi.

[47] Franklin SS, Thijs L, Hansen TW, Li Y, Boggia J, Kikuya M, Björklund-Bodegård K, Ohkubo T, Jeppesen J, Torp-Pedersen C, Dolan E, Kuznetsova T, Stolarz-Skrzypek K, International Database on Ambulatory Blood Pressure in Relation to Cardiovascular Outcomes Investigators (2012). Significance of white-coat hypertension in older persons with isolated systolic hypertension: a meta-analysis using the International Database on Ambulatory Blood Pressure Monitoring in Relation to Cardiovascular Outcomes population. Hypertension.

